# Robotics in Nursing: Protocol for a Scoping Review

**DOI:** 10.2196/50626

**Published:** 2023-11-13

**Authors:** Elizabeth Mirekuwaa Darko, Manal Kleib, Gillian Lemermeyer, Mahdi Tavakoli

**Affiliations:** 1 Faculty of Nursing College of Health Sciences University of Alberta Edmonton, AB Canada; 2 College of Natural and Applied Sciences, Electrical and Computer Engineering University of Alberta Edmonton, AB Canada

**Keywords:** automation, robots, nursing, nursing robots, nursing robotic technologies

## Abstract

**Background:**

Globally, health care systems are challenged with the shortage of health care professionals, particularly nurses. The decline in the nursing workforce is primarily attributed to an aging population, increased demand for health care services, and a shortage of qualified nurses. Stressful working conditions have also increased the physical and emotional demands and perceptions of burnout, leading to attrition among nurses. Robotics has the potential to alleviate some of the workforce challenges by augmenting and supporting nurses in their roles; however, the impact of robotics on nurses is an understudied topic, and limited literature exists.

**Objective:**

We aim to understand the extent and type of evidence in relation to robotics integration in nursing practice.

**Methods:**

The Joanna Briggs Institute methodology and the PRISMA-ScR (Preferred Reporting Items for Systematic Reviews and Meta-Analyses extension for Scoping Reviews) checklist will guide the scoping review. The MEDLINE (Ovid), Embase (Ovid), CINAHL Plus with Full Text (EBSCOhost), Scopus, Cochrane Library, and IEEE Xplore electronic bibliographic databases will be searched to retrieve papers. In addition, gray literature sources, including Google Scholar, dissertations, theses, registries, blogs, and relevant organizational websites will be searched. Furthermore, the reference lists of included studies retrieved from the databases and the gray literature will be hand-searched to ensure relevant papers are not missed. In total, 2 reviewers will independently screen retrieve papers at each stage of the screening process and independently extract data from the included studies. A third reviewer will be consulted to help decision-making if conflicts arise. Data analysis will be completed using both descriptive statistics and content analysis. The results will be presented using tabular and narrative formats.

**Results:**

The review is expected to describe the current evidence on the integration and impact of robots and robotics into nursing clinical practice, provide insights into the current state and knowledge gaps, identify a direction for future research, and inform policy and practice. The authors expect to begin the data searches in late January 2024.

**Conclusions:**

The robotics industry is evolving rapidly, providing different solutions that promise to revamp health care delivery with possible improvements to nursing practice. This review protocol outlines the steps proposed to systematically investigate this topic and provides an opportunity for more insights from scholars and researchers working in the field.

**International Registered Report Identifier (IRRID):**

PRR1-10.2196/50626

## Introduction

### Background

Globally, health care systems are challenged with the shortage of health care professionals, particularly nurses. Nurses and midwives account for nearly 50% of the global health care workforce [1], with the same proportion bearing out in the Canadian health care system [2]. It has been projected that by 2035, there will be a global deficit of 12.9 million health care professionals, with nurses and midwives making up half of the health care workforce shortage [1,3]. Several factors account for the increasing decline in the nursing workforce, including increased workload, insufficient resources, chronic burnout, stress, early retirement, understaffing, job dissatisfaction, work-related and musculoskeletal injuries, and low remuneration [4-10]. Other factors include post–COVID-19 discontentment, disease and patient acuity increase, and the aging population and workforce [4,7,11,12]. The COVID-19 pandemic exacerbated the already existing nursing shortage crisis.

Nurses undertake multiple and complex activities within any care setting, and their most central role is to provide efficient and effective patient-centric care [13]. Research shows increased staffing leads to greater job satisfaction and retention, reduced mortality and associated injuries among nurses and patients [14]. Nonetheless, about one-third of nurses' time is often spent on administrative and nonpatient care activities and auxiliary tasks that could be completed by other staff, such as nursing aides, assistants, or administrative staff [15,16]. Among health care professions, nurses are ranked with the highest prevalence of musculoskeletal pain and injuries [17] and an annual prevalence of work-related musculoskeletal diseases of 77.2% [18]. The results from these injuries are absenteeism, sick leaves, health-related problems, and disabilities, affecting the quality of health and putting added pressure on the health care workforce [19]. In addition, there is a significant rise in error rates among nurses resulting from physical and mental exhaustion, compromising patient and nurses' safety [20]. Nurses' time and skills are highly valuable, as they have specialized knowledge essential to achieving optimal health outcomes. Identifying alternative solutions that can decrease nurses' time lost in undertaking noncritical tasks is therefore necessary. Robotics is 1 solution that is gaining growing attention due to the many activities that robots can undertake to support nursing practice, strengthen health care delivery, improve overall health outcomes and the quality of care, and fill current and projected gaps in the workforce needed to meet population health needs [21]. When thoughtfully and appropriately integrated, robotics could also save money for the health care system and free up time for nurses to focus on patient care, thereby mitigating negative impacts on patients and the mental well-being of nurses [22]. On the other hand, robotics integration in nursing also raises inquiries regarding the methodologies, technologies, and ethics of developing robots to support the work of nurses and other health care professionals [23].

In broad terms, robots in health care are classified into surgical, rehabilitation, medical assistants, and hospital service robots [24]. These robots are referred to as assistive robots because they provide users with assistance and are subcategorized as physically and socially assistive robots [25,26]. Physically assistive robots are those robots that assist end users through physical interaction and may be designed to perform tasks without much physical human input, such as exoskeletons, intelligent wheelchairs, robotic manipulators, and walking assistive robots [26-30]. On the other hand, socially assistive robots are physical or digital entities capable of social interaction functions that promote users' psychological, emotional, cognitive, and overall well-being [26,31,32]. Socially assistive robots, such as Nao and Pepper robots, are used to provide affective therapy, social facilitation, companionship, and physiological therapy in patients [28,30,33]. The socially assistive robots have proved useful in caring for older people, assisting with activities of daily living such as medication reminders, undertaking household tasks, health monitoring to enhance safety, supporting independence and aging in place, and overall users' well-being [34]. Multimedia Appendix 1 provides a visual representation of classifications of the different types of robots used in health care.

The boundaries between the areas of application of robots in nursing are somewhat fluid. Different names are given to robots used by nurses in health care facilities, such as nurse assistants, hospital service robots, delivery care robots, and nurse robots. These names are based on their specification and practical use in care settings. For this review, the robots used in the nursing practice will be referred to as nursing robots. Nursing robots combine the features and functions of medical assistant robots and hospital service robots. The nursing robots are specifically designed to perform activities to supplement nurses' work at hospitals, long-term facilities, and home care [26]. These robots are socially and physically assistive and can be categorized into humanoids and nonhumanoid robots, including telepresence robots. Since nursing robots can provide assistance with complex activities within care settings, they can support nurses in providing services to patients unrelated to medical functions [24].

The surge in the interest in using nursing robots in health care is because of the technological capacity to strengthen health care delivery and improve quality health outcomes [21]. Nursing robots can complement nurses' efforts by undertaking repetitive work such as retrieving and delivering supplies, providing ambulation services, medication delivery services, assisting in patient transfers, lifting, and assisting with standardized approaches to care management [30,35,36]. In addition, robots can enhance nurses' work by monitoring patients' condition and vital signs and providing patient reminders [26,31]. Furthermore, nursing robots can serve as frontline actors during emergency response and routine care to prevent or reduce nurses' physical contact and exposure to hazardous substances, infections, or contamination. These tasks are usually physically and mentally demanding. They are routine activities that can be assigned to a nonhuman entity, thereby reducing the physical and mental stress for nurses, saving money for the health care system, and freeing up nurses’ time to focus on patient care [22]. To this end, nursing robots can decrease the physical demands of nurses' work and reduce the rate of injuries and human errors, thereby increasing job satisfaction and improving patient care quality and outcomes [37,38]. For nurses, incorporating nursing robots in health care means improved workflow, increased work quality and productivity, and reduced workload. To health systems, robots could be an ideal solution to the wicked problem of nurse shortage.

To identify published literature reviews on the use of robotics or robots in nursing practice, a preliminary literature search was conducted (April 13, 2023) in CINAHL Plus with Full Text database, and 4 reviews were located. Ohneberg et al [35] focused their review on assistive robotic systems and their application areas in nursing practice. The other reviews focused on identifying the different tracks in which robots are used in nursing, barriers to implementation, and the outcomes of robots and other automated devices on nurses' activities [31,37,39]. Based on the findings from these reviews, the authors proposed that further research studies are needed to explore the acceptance and impact of robotics systems on nursing practice and examine facilitating factors and ethical concerns of implementing robots [35]. In addition, the authors suggested examining research designs used for undertaking studies in nursing robotics to ascertain the consistency and rigour of the quality of the studies [39] and evaluate which devices and functions will improve and support nurses' work [37]. Further, the authors noted the need to examine the human-robot interactions, focusing on monitoring robots and exploring psychological barriers that need to be overcome to influence and increase the acceptance of robots [31].

Despite the 4 reviews published on this topic, there remains a pressing need to map out the extant literature to better understand the current research discourses on the influence of robots (regardless of their type) on nurses and nursing practices across clinical care settings. A clear picture of what has and has not been attended to in terms of both (1) the robot's ability to reshape, reconstitute, and reconvene nursing practices, as well as (2) their influence on the nurse's mental, physical, and overall well-being is necessary to generate and inform future research. Scoping reviews are appropriate for examining emerging areas where little is known about a phenomenon of interest [40]. Broadly, this scoping review aims to understand the extent and type of evidence in relation to robotics integration in nursing practice. More specifically, the review will (1) identify the available evidence reporting on robots used in supplementing nursing and their related tasks in direct clinical care, (2) identify the benefits and challenges associated with their use, and (3) understand nurses' perceptions of the impact of robots on their work, and patient and clinical care.

### Research Questions

The overarching question guiding this review is, what is the current evidence on the integration and impact of robots and robotics into direct nursing care? The following subquestions will be examined: (1) what are the types of robots and robotic technologies available to support nursing care? (2) What is the range of tasks (nursing and nonnursing related) that robots and robotic technologies can assist nurses with in providing direct clinical care? (3) What are the benefits and challenges associated with robotic integration in nursing? (4) What are nurses’ perceptions and views on the impacts of robots and robotic technologies on nurses’ work, patient care, and health systems that can be achieved when robots are used to supplement nursing practice from process, structure, and outcome perspectives?

## Methods

### Study Design

The Joanna Briggs Institute methodology and the PRISMA-ScR (Preferred Reporting Items for Systematic Reviews and Meta-Analyses extension for Scoping Reviews) checklist for scoping reviews will guide the scoping process [41,42]. We will review existing literature, examine the extent, scope, and nature of research activities to identify gaps and summarize and disseminate research findings [42]. An initial search (January 05, 2023) in the Open Science Framework revealed that no study of this nature had been registered; hence, this review will be registered in the Open Science Framework registry, which is a platform where researchers register prospective scoping reviews to inform other researchers interested in undertaking similar work, hence minimizing the risk for duplication.

### Inclusion Criteria

The Joanna Briggs Institute's participants, concept, and context framework will be applied to guide the decision-making about the inclusion and exclusion of studies for the review, eliminate studies that are not essential to the central research questions, and maintain the consistency of the review process [42].

### Participants

This review will focus on nurses working in different roles, including licensed practical nurses, registered practical nurses, registered nurses, registered psychiatric nurses, community health nurses, and nurse practitioners providing direct care within a variety of practice settings. Unless nurses are included as part of the study population, studies that report on other health professionals and patients will be excluded because they are not the participants of interest.

### Concept

The authors recognize the differences in the classification of nursing robots; therefore, all papers that report on the use of nursing robots used in nursing practice, irrespective of the reported outcomes or the clinical context, will be included. In addition, any paper that does not report on robot use within the nursing practice or the use of socially assistive robots that focus on patient care will be excluded.

### Context

This review will consider studies that examine robot use in all nursing practice settings, including long-term, primary care, acute care, rehabilitation, community care, and health care settings. In addition, the review will not be limited to any geographical location as the authors want to evaluate nursing robots from an international perspective and be comprehensive in the search.

### Types of Sources

This review will consider all study types, including qualitative, quantitative, and mixed methods research, except for published reviews on the topic, including systematic reviews, scoping reviews, narrative or traditional literature reviews, meta-analyses, and integrative reviews. Excluded reviews may be used later for discussion and comparison of results. In addition, studies that report on theoretical and methodological approaches will also be examined for relevance and considered for inclusion. Gray literature in the form of guidelines, theses, dissertations, discussion papers, white papers, reports, brief reports, specific guidelines, and unpublished papers that report on nursing robots or robotic technologies in nursing practice will be included. Blogs and internet research sites that report on the piloting of robots relevant to nursing practice will also be considered for inclusion. Conference proceedings and publications, opinion pieces and commentaries will be excluded as they might not provide in-depth information for analysis. Studies will be limited to the English language due to the lack of funding to support the translation of the materials, and reviewers can only speak English. Since the area of robotics in nursing is novel and evolving, the year of publication will be from the beginning of indexed papers in databases to the current search date. This will enable an assessment of the development of robots or robotics in nursing practice and their evolution over time.

An eligibility form for screening the papers will be used to guide the screening process (Multimedia Appendix 2), making the screening process clearer to the reviewers and maintaining the robustness of decision-making regarding the inclusion and exclusion of studies [43]. In total, 2 reviewers will independently screen data using the eligibility form provided and record the results. The kappa statistic, sometimes called Cohen kappa, a robust statistical method used most frequently, will be used to measure interrater reliability between the reviewers [43,44]. The kappa statistic method is applicable when two or more reviewers make decisions at various points in a study's screening and data extraction process [43,44]. Interrater reliability is important to ensure that data collected in the study are correct depictions of the variables measured [43].

### Search Strategy

A comprehensive search strategy will be developed using predefined search terms in consultation with a health science librarian. A search was conducted in 1 database (CINAHL Plus with Full Text) to determine the appropriateness and effectiveness of the search terms and strategy (Multimedia Appendix 3) proposed for this review on September 26, 2023. The following Boolean operators and keywords proposed and identified will guide the search of the published and unpublished (grey literature) relevant to the topic: Automat* OR Robot* AND Nurs* AND Patient* OR In-patient* OR Hospital* OR "Nursing Care" OR "Clinical Care" OR "Direct Care," in four electronic bibliographic health databases, one interdisciplinary database and one electronic engineering database: Medline (Ovid), EMBASE (Ovid), Cumulative Index to Nursing and Allied Health Literature (CINAHL) Plus with Full Text (EBSCOhost), Scopus, Cochrane Library, and IEEE Xplore. The health science databases are appropriate for researching the topic because nursing papers are published in these databases. Whereas the IEEE Xplore database publishes papers from computer science, electrical engineering, and other technological disciplines, nursing robots are found in this database. The search strategy will be further refined and adapted to the remaining databases as necessary. In addition, the reference lists of all included studies retrieved from studies databases, and gray literature will be checked to ensure relevant papers are not missed.

For the gray literature, Google Scholar, dissertations, theses, registries, blogs, and relevant organizational websites will be searched to identify papers relevant to nursing robots. The gray literature provides studies that might not have been peer-reviewed or indexed in databases, thereby providing balanced evidence on the subject matter. Finally, the search strategy will use an iterative process until the search becomes robust to retrieve all related papers. The authors expect to begin the data searches in late January 2024.

### Study Selection

The PRISMA-ScR checklist will be used as a guide in reporting the scoping review [45]. In addition, database search results will be exported to the Mendeley reference manager to keep track of search results, organize references and bibliographies, and facilitate collaboration among all authors [46]. Retrieved studies will then be exported into Covidence Management Software (Covidence) to remove duplicates and support organizing and screening the studies into a PRISMA (Preferred Reporting Items for Systematic Reviews and Meta-Analyses) chart.

In total, 2 reviewers (EMD and GL) will screen the studies independently at each stage of the screening process. Stage 1 involves the title and abstract screening, where eligible studies are passed to stage 2 for full-text screening. In stage 2, the developed eligibility criteria form will be used to assess the eligibility of the papers for either inclusion or exclusion. Studies marked for exclusion will be assigned a reason for the exclusion to help keep track of and report on them in the findings. When conflicts arise due to disagreements between reviewers on studies during the full-screening stage, a third reviewer (MK), a content expert, will be invited to help resolve the conflicts. An interlibrary loan request will be sent to the library if full-text papers cannot be retrieved. Further, if possible, a request will be sent to the authors or journals to obtain more information about a study, as needed and feasible. If attempts made to retrieve papers are unsuccessful, the studies will be excluded and documented in the PRISMA diagram and reported in the findings.

### Data Extraction

An Excel (Microsoft Corp) sheet extraction table will be developed and piloted to record essential information in the included studies to allow for transparency and clarity among reviewers. Each reviewer will independently extract the following data characteristics from the included studies grouped under the participants, concept, and context domains: study citation details (author's name, year of publication, country of publication, and title), study design, study purpose, participants (population and sample size), context (study settings), concept (types and range of tasks undertaken by the robots, benefits and challenges, nurses' views, and perceptions), and outcomes (Multimedia Appendix 4). The identified extracted characteristics will support answering the research questions and in identifying the gaps in the literature. The extraction template is iterative; hence, it will be piloted on n=10 of the papers and may undergo refinement during the review and extraction stages to capture information based on how researchers reported information.

In total, 2 reviewers (EMD and GL) will undertake the data extraction process independently to reduce the chance of bias and errors [41]. Data extraction information will be compared for agreements and differences. In the event of discrepancies among the reviewers, a consensus will be reached by inviting a third reviewer (MK) and having a discussion for reconciliation. In scoping reviews, missing data are not usually dealt with, but the authors will decide how to proceed where possible. Scoping reviews do not aim to assess the quality of evidence, the robustness, or the generality of the evidence [40] but rather to identify literature that addresses the topic of interest. Therefore, the study quality will not be appraised in this scoping review.

### Data Analysis

Data in this review will be systematically examined to minimize bias in interpreting the information abstracted from the included studies. Basic descriptive statistics will be applied to describe, aggregate, and report the findings in numerical values to enable a comprehensive evaluation of study characteristics such as the geographic distribution of included studies, populations studied, and publication time lines [47,48]. Content analysis will be applied to capture relevant information from each individual study to answer the research questions of interest in this review [49,50]. All team members will be involved in the data analysis to ensure a robust and timely analytical process.

## Results

Information related to the characteristics of the included studies will be reported in the PRISMA chart to provide an overview of the body of the literature available, included and excluded studies, and the data sources used. This will be followed by a description of the characteristics of the included studies, using both a narrative and a tabular format. Graphs may also be used where appropriate to further illustrate the findings visually. A list of the excluded studies will also be provided with reasons for exclusion. To answer research questions 1 and 2, data related to the types of robots and robotic technologies available to support nursing care, the names of those robots and the different terminologies used to describe them in the literature, and the different tasks that these robots can assist nurses with will be reported in a tabular format to map out this literature. To answer research questions 3 and 4, abstractions from each included study in relation to the benefits and challenges associated with robotics integration, nurses’ perceptions, and views on the impacts of robots and robotic technologies on nurses’ work, patient care, and health systems will be summarized and reported narratively. Specific outcomes discussed in the literature will be noted and further organized according to the conceptual framework (structure, process, and outcome) proposed by Donabedian to facilitate a synthesis of these findings [51,52] to answer the research questions.

## Discussion

### Principal Findings

This scoping review aims to understand the extent and type of evidence in relation to robotics integration in nursing practice. Prior work has focused on investigating assistive robotic systems and their application areas in nursing practice [35]. Also, some reviews have identified the different tracks in which robots are used in nursing, barriers to implementation, and the outcomes of robots and other automated devices on nurses' activities [31,37,39]. The findings from these reviews reveal more research is needed to explore other areas of robotics in nursing practice, including human-robot interactions, and evaluating the robotics that supports nurse’s work [31,35,37]. Building on the evidence, this review will (1) identify the available evidence on robots being used to supplement nursing and their related tasks in direct clinical care, (2) identify the benefits and challenges associated with their use, and (3) understand nurses' perceptions of the impact of robots on their work, patient care, and the health system.

The results from this review will be discussed in detail and in alignment with each research question. Furthermore, the results will be compared against what is already reported in the literature about robotics in nursing to help address the identified knowledge gaps and reveal new areas of research that have yet to be explored. In addition, other relevant literature sources, such as publications about digital health and health service research, will also be reviewed and used where appropriate. The discussion will also include detailed recommendations and implications relevant to nursing practice, education, research, and policy. The findings will also be published in a journal and disseminated at conferences, seminars, workshops, and web-based platforms.

### Limitations

Only papers written in English will be included; hence, there is a possibility of missing out on other papers written in other languages. Furthermore, despite the comprehensive and extensive inclusion criteria, there is also the possibility of missing some relevant sources of information. Since the included studies will not be critically appraised, the rigor and trustworthiness of included studies cannot be ascertained; however, this scoping review may help provide a general assessment of the overall quality of research available and outline areas of research warranting further investigation.

### Conclusions

This scoping review aims to investigate the current evidence on the integration and impact of robots and robotics nursing. By conducting a comprehensive search of peer-reviewed and nonpeer-reviewed sources, this scoping review will systematically map out and describe the current evidence about nursing robots and add to the body of knowledge on robots' integration in nursing. The scoping review is an appropriate method because the searches and inclusion of evidence are broad and unlimited to investigate an emerging area of science, identify knowledge gaps, and provide a direction for future research. Considering the novelty of robotics integration in health care, the findings from this review will be instrumental in expanding the scholarly discourse about robotics as one of the emerging technologies that will have significant implications for patient care and nursing practice in the short and long term. This review protocol outlined the steps proposed to systematically investigate this topic and provide an opportunity to gain more insights on conducting the proposed review from scholars and researchers working in the field.

## Appendix

Multimedia Appendix 1 Classification of the different types of health care robots.

Multimedia Appendix 2 Robotics in nursing: a scoping review eligibility form.

Multimedia Appendix 3 Search strategy.

Multimedia Appendix 4 Extraction table: characteristics of included studies.

## Abbreviations

PRISMA: Preferred Reporting Items for Systematic Reviews and Meta-Analyses
PRISMA-ScR: Preferred Reporting Items for Systematic Reviews and Meta-Analyses extension for Scoping Reviews

## Data Availability

All data used in this review, including the extraction table, the search strategy, and eligibility criteria, will be attached as appendices in the final paper. Any other document will be made available upon request to the corresponding author.
