# Supplementary material for: Robotics in Nursing: Protocol for a Scoping Review
Source: JMIR Res Protoc. 2023 Nov 13;12:e50626. doi: 10.2196/50626 (PMC10682918; doi:10.2196/50626)
Supplement: Multimedia Appendix 1 [file resprot_v12i1e50626_app1.doc]

**Multimedia Appendix 1.** Classification of the different types of robots in health care.
