# Supplementary material for: Robotics in Nursing: Protocol for a Scoping Review
Source: JMIR Res Protoc. 2023 Nov 13;12:e50626. doi: 10.2196/50626 (PMC10682918; doi:10.2196/50626)
Supplement: Multimedia Appendix 2 [file resprot_v12i1e50626_app2.doc]

**Multimedia Appendix 2**. Robotics in nursing: a scoping review eligibility form.

| Study ID (Author year) |  | | |
| --- | --- | --- | --- |
| Title |  | | |
| Journal |  | | |
| Extractor |  | | |
| Eligibility criteria | | | |
| A. Design | | | |
| Has a specified study design | | | |
| Yes → Include | No → Exclude | | Unclear |
| B. Participants | | | |
| Nurses (LPNs, **RPN**s**,** RNs, RPNs, CHNs, & NPs) | | | |
| Yes → Include | No → Exclude | | Unclear |
| C.   1. Does the study describe robots in a nursing practice environment?   or   1. Does the study specify the settings/ unit where the robot is used in nursing practice? | | | |
| If any above Yes → Include | | None of the above No → Exclude | Unclear |
| D. Does the study report on the outcome of nursing robots' incorporation into nursing practices? | | | |
| Yes → Include | No → Exclude | | Unclear |
| Final decision | | | |
| Include | Exclude | | Unclear |
| If unclear, further action taken: | | | |
