# Supplementary material for: Robotics in Nursing: Protocol for a Scoping Review
Source: JMIR Res Protoc. 2023 Nov 13;12:e50626. doi: 10.2196/50626 (PMC10682918; doi:10.2196/50626)
Supplement: Multimedia Appendix 3 [file resprot_v12i1e50626_app3.doc]

**Multimedia Appendix 3.** Search strategy.

CINAHL Plus with full text

Date: September 27, 2023

Total Number of Hits: 2,345

| Search ID# | Search Terms | Results |
| --- | --- | --- |
| S1 | Automat* OR Robot* | 72,776 |
| S2 | Nurs* | 1,014,215 |
| S3 | Patient* OR In-patient* OR Hospital* OR "Nursing Care" OR "Clinical Care" OR "Direct Care | 2,835,174 |
| S4 | S1 AND S2 AND S3 | 2,480 |
| S5 | S1 AND S2 AND S3  Limiter: English language | 2,345 |
