# Supplementary material for: Robotics in Nursing: Protocol for a Scoping Review
Source: JMIR Res Protoc. 2023 Nov 13;12:e50626. doi: 10.2196/50626 (PMC10682918; doi:10.2196/50626)
Supplement: Multimedia Appendix 4 [file resprot_v12i1e50626_app4.doc]

**Multimedia Appendix 4**. Extraction table: characteristics of included studies.

| **Study characteristics** | **Study 1** | **Study 2** |
| --- | --- | --- |
| Study citation details (authors, date, country, & title) |  |  |
| Study design |  |  |
| Study aims or purpose |  |  |
| **Participants:**   - Licensed Practical Nurses (LPNs)**,** Registered Practical Nurses (RPNs)**,** Registered Nurses (RNs), Registered Psychiatric Nurses (RPNs), Community Health Nurses (CHNs), and Nurse Practitioners (NPs). - Population size |  |  |
| **Context:** Long-term, primary care, acute care, rehabilitation, and community care settings, nursing practice, and any country |  |  |
| **Concept:** Nursing robots with regards to:   1. the types of robots available in nursing care. 2. the range of tasks (nursing and non-nursing related) that robots and robotic technologies can assist nurses in direct clinical care with. 3. the benefits and challenges associated with robotic integration in nursing. 4. nurses’ perceptions and views on the impacts of robots and robotic technologies on nurses’ work, patients', clinical care and health systems that can be achieved when robots are used to supplement nursing care from a process, structure, and outcomes perspective? |  |  |
| **Outcomes:** the reported outcomes. |  |  |
